# Assessing the Relationship Between Sleep Duration and the Prevalence of Chronic Kidney Disease Among Veterans in the United States: A 2022 Behavior Risk Factor Surveillance System (BRFSS) Cross-Sectional Study

**DOI:** 10.7759/cureus.68538

**Published:** 2024-09-03

**Authors:** Francis Okeke, Uyonne T Ugwuoke

**Affiliations:** 1 Department of Medical Informatics, The University of Oklahoma Health Sciences Center, Tulsa, USA; 2 Department of Emergency Medicine, North Knoxville Medical Center, Knoxville, USA

**Keywords:** cdc brfss, veterans, sleep duration, chronic kidney disease, prevalence

## Abstract

Introduction: Chronic kidney disease (CKD) is more prevalent among veterans in the United States than in the general population. Similarly, veterans also exhibit higher rates of abnormal sleep duration compared to the general population. The aim of this study was to investigate the association between self-reported length of sleep and the prevalence of CKD among veterans in the United States using responses from the 2022 Behavioral Risk Factor Surveillance System (BRFSS).

Methods: For this cross-sectional study, a total of 53,211 veterans who responded to the 2022 BRFSS survey were analyzed. Measures include the outcome variable which is self-reported CKD diagnosis and a major independent variable sleep duration. Sleep duration was recategorized into ≤ 5 hours (short sleep duration), 6-10 hours (normal sleep duration), and >10 hours (long sleep duration). Covariates included gender, age, race, residence, insurance, alcohol consumption, diabetes comorbidity, coronary artery disease (CAD) comorbidity, and stroke comorbidity. Descriptive, bivariate, and multivariate logistic regressions were conducted using the SAS software (SAS Institute Inc., Cary, North Carolina, United States).

Results: The prevalence of CKD among veterans in the United States is 3332 (6.29%). Veterans with sleep duration of 6-10 hours had 17.5% lower odds of CKD than veterans who slept for ≤5 hours (adjusted OR (AOR)= 0.825, 95%CI= 0.821-0.830; P=<0.0001). Veterans who slept for more than 10 hours had 68.2% higher odds of having CKD (AOR=1.682, 95%CI= 1.662-1.702; P=<0.0001). Additionally, veterans diagnosed with diabetes, stroke, and coronary artery disease had 2.447-2.103, and 2.838, respectively, higher odds of developing CKD (AOR=2.447, 95%CI= 2.435-2.459; p=<0.0001). Veterans who were 65 years and older had higher odds of developing CKD compared to those aged 35-44 years (AOR= 5.743, 95%CI= 5.669-5.818; P<0.001). The odds of having CKD were also higher among veterans who identified as Black (AOR 1.397, 95%CI =1.388-1.405; P<0.01) or as Hispanic (AOR =1.318, 95%CI = 1.307-1.329; P<0.01) compared to non-Hispanic White veterans. Those who identified as Asian had lower odds of CKD (AOR= 0.87, 95%CI=0.853-0.888; P<0.01). Furthermore, veterans who consumed alcohol had 7.8% lower odds of having CKD as compared to individuals who did not consume alcohol (AOR= 0.922, 95%CI =0.918-0.927; p=<0.0001). Male veterans had 24.7% lower odds of having CKD as compared to female veterans (AOR = 0.753, 95%CI= 0.747-0.758; P<0.001).

Conclusion: This research provides evidence of a greater prevalence of CKD among veterans with short sleep duration (≤ 5 hours) and long sleep duration (> 10 hours). Sleep hygiene education and sleep optimization programs can improve sleep and boost overall kidney health among veterans.

## Introduction

Chronic kidney disease (CKD) ranks among the leading causes of death in the United States (US) [1]. As of 2023, there were approximately 36 million people with CKD in the United States [2]. An estimated one in six veterans suffer from CKD [2,3], an increase of almost 35% over its prevalence in the general population [4]. The treatment of veterans with CKD incurred nearly $20 billion in costs 10 years ago for the Department of Veterans Affairs (VA) excluding expenses for advanced cases necessitating kidney replacement therapy [5,6]. This expenditure is expected to have significantly increased, considering the VA's projection of over 29,500 new CKD diagnoses among veterans annually [5]. A recent study examined historical data from individuals diagnosed with CKD within the Military Health Service, findings revealed that CKD was more prevalent among older veterans (aged over 65 years), males, and individuals of African American descent, and less common among those serving in active-duty military roles [7]. Among the US veteran population totaling 18.3 million individuals, 16.2 million are male [8]. Additionally, 74% of veterans identify as non-Hispanic White, while 72% are above the age of 50 [8,9].

Adequate sleep is crucial for maintaining normal bodily functions [10,11]. The American Academy of Sleep Medicine and the Sleep Research Society recommend adults aged 18-60 years aim for 7-9 hours of sleep per night to support overall health [12,13]. Sufficient sleep has gained significant attention, highlighted in the Healthy People 2030 goals [14], and emphasized by the American Heart Association in its Updated Life's Essential 8 as an important measure for cardiovascular health [11]. However, data from the 2020 Behavioral Risk Factor Surveillance System (BRFSS) reveals that 33% of adults in the US report insufficient sleep, defined as less than seven hours per day [15]. This shortfall in sleep results in an annual economic loss exceeding $400 billion and accounts for approximately 1.25 million lost working days due to sleep deprivation among workers [16,17]. The increased popularity of social network sites, cyberspace technology [11], and heightened levels of perceived stress significantly contribute to sleep issues among American adults [18]. Prior studies on sleep dynamics among US service members reveal that a significant portion of them consistently report insufficient daily sleep below seven hours [12,19-22]. Various social and environmental factors such as safety, noise levels, physical surroundings, and neighborhood characteristics can significantly impact sleep duration [15,22]. Studies link insufficient sleep among veterans to both reducing personnel size and psychosocial factors such as irregular shift hours, fluctuating environmental conditions, stress from frequent deployment, and anxiety due to family separation [12,19-21,23]. Similar high trends of self-reported insufficient sleep were noted among civilian adults and force members aged 25-44, males, non-Hispanic Black individuals, low-income earners, those with no or minimal formal education [12,15], and those residing in the southeastern states and the Appalachian region [15,24]. Current or former smokers in the military are more likely to report insufficient sleep compared to non-smokers. Additionally, individuals serving in the Army, Marine Corps, and Navy were more likely to report short sleep duration compared to those in the Air Force [12].

Prolonged inadequate sleep (<6 hours of sleep per day) is associated with a plethora of health problems [12,19,22,25-27], including increased risk for obesity, hypertension, diabetes mellitus, and depression [12,28]. While the connection between prolonged diabetes mellitus and hypertension and the development of CKD is well-established, there remains limited understanding regarding the relationship between sleep duration and CKD. Previous studies have described the association between sleep duration and kidney disease as u-shaped [29,30], suggesting similar outcomes for both individuals sleeping less than five hours per day and those sleeping more than 10 hours per day. However, the direct mechanism underlying either short or prolonged sleep remains unclear. 

The human circadian clock plays a vital role in regulating physiological functions of the kidney, such as blood pressure and glomerular filtration rate (GFR) [10,26,31]. Military personnel often experience irregular sleep patterns and disruptions in their circadian cycles [21]. These disturbances can trigger the release of stress hormones such as cortisol and catecholamines [32]. Prolonged exposure to these hormones may contribute to the progression of kidney dysfunction. Additionally, getting too little sleep can result in insufficient hydration [33], as well as increased activity of the sympathetic nervous system, and diminish the natural decline in blood pressure that occurs during sleep [10,11,31]. Furthermore, sleep limitation to only four to five hours in a day for close to a week is capable of causing reduced glucose tolerance and insulin sensitivity [12,25]. In general, the mechanism through which insufficient sleep affects vascular response and glucose regulation involves chronic inflammation and free radical formation causing oxidative stress on body cells and immune systems [11,25,34].

Both brief and extended duration of sleep have been linked with the possibility of developing obesity, heart problems [35], diabetes mellitus [25], and general poor health outcomes [35,36]. A significant portion of the US veteran population is obese [37], with prior studies attributing this to irregular eating habits, trauma, physical immobility, and psychological disorders [23,37]. Obesity also increases the likelihood of conditions such as obstructive sleep apnea (OSA), hypertension, type 2 diabetes mellitus, and depression [28,37]. A recent study reported over 50% of surveyed veterans with heart and vascular disease risk having sleep apnea [38]. OSA disrupts breathing during sleep, resulting in reduced oxygen levels in the blood [31,39]. This can lead to increased blood pressure and proteinuria, ultimately reducing the glomerular filtration rate over time [31,39,40]. Consequently, this presents another pathway for the progression of CKD.

In the 2022 BRFSS survey, participants asked about their average sleep duration over a 24-hour period reported a range of 1-24 hours [41]. The CDC defines adequate sleep based on age requirements, with seven hours per day serving as the cutoff [42]. However, previous literature has further categorized sleep into short (less than five hours per day), hours per day), adequate (6-9 hours per day), or prolonged (>9 hours per day) durations. This classification provides a precise way to associate individual sleep patterns with various health outcomes [35].

This article was previously presented as a meeting abstract at the 2024 Appalachian Student Research Forum on April 5, 2024.

## Materials and methods

This study utilized responses from the 2022 BRFSS data set. The BRFSS is a yearly CDC-assisted health-data collection survey comprising telephone surveys of adults (18 years and above) in all 50 states, US territories, and the District of Columbia [23,43]. Most of the BRFSS interviews conducted in 2022 were via cell phone. Response rates, cooperation rates, and refusal rates for BRFSS are calculated using standards set by the American Association for Public Opinion Research (AAPOR) [43]. The data set for the 2022 survey comprised 445,132 observations and the median survey response rate was 45.1% [43]. The BRFSS is publicly available, de-identified data that does not require an institutional review board approval or an exempt status before usage.

This study included all veterans with a sample size of 53211, which represents 12% of the entire respondent population. The outcome variable (CHCKDNY2) described as “Ever told you have a kidney disease” was recorded as CKD into categorical variables "Yes" and "No". The independent variable (SLEPTIM1) which asked, "On average, how many hours of sleep do you get in 24 hours?" was categorized into three ordinal groups: Short sleep duration (≤ 5 hours), Normal sleep duration (6-10 hours), and Long sleep duration (>10 hours).

Covariates included gender, age, race, residence, insurance, alcohol consumption, diabetes comorbidity, stroke comorbidity, and coronary artery disease (CAD) comorbidity. The age variable (_AGE_G) was retained as discrete without recoding, structured into six imputed age categories at 10-year intervals, starting from 18 years and continuing to 65 years and above (1 = age 18 to 24, 2 = age 25 to 34, 3 = age 35 to 44, 4 = age 45 to 54, 5 = age 55 to 64, 6 = age 65 or older). The first category spans a six-year interval (18-24 years). The gender variable (SEXVAR) and race variable (_IMPRACE) remained binary and nominal respectively without recoding. The variables for insurance (_HLTHPLN), alcohol (DRNKANY6), diabetes comorbidity (DIABETE4), stroke comorbidity (CVDSTRK3), and CAD comorbidity (CVDCRHD4) were recoded into categorical variables, while residence (_URBSTAT) was recoded into a nominal variable. 

All analyses were performed with SAS version 9.4 (SAS Institute Inc., Cary, North Carolina, United States). Weight, cluster, and strata measures from the BRFSS codebook were applied to output weighted statistics of variables in relation to veterans with CKD. Bivariate analysis was done using logistic regression to calculate unadjusted odds ratios (ORs) and 95% confidence intervals (CIs) between veterans with CKD and sleep duration as well as with various variables. Variables that met a cut-off of p-value ≤0.2 were selected for further analysis. Multivariate analyses were conducted to calculate ORs with 95% CIs for the association between veterans with CKD and all variables. The multivariate regression model was also applied to check interactions between the variables. P < 0.05 was used to indicate final statistically significant results.

## Results

Descriptive analysis

The sample size was 53,211. Of this, 3332 (6.29%) veterans had CKD. Of the 3332 veterans with CKD, the majority comprised veterans who slept for 6-10 hours (n=1746, 71.83%) as compared to veterans who averaged about ≤ 5 hours of sleep (n=464, 22.40%) and those who slept for more than 10 hours (n=100, 5.77%). Most respondents with CKD were male veterans (n=3028, 88.91%) and most were aged 65 years and older (n=2665, 70.93%). Regarding racial description, White ethnicity comprised the majority (n=2725, 70.67%) as compared to Black (n=252, 14.61%) and Hispanic (n=160, 9.30%) ethnicity. Additionally, of the veterans with CKD, 45% also had diabetes, 19.87% had stroke, and 28.96% had depression. Table 1 provides the breakdown statistics for the variables.

**Table 1:** Sample characteristics and distribution of variables among veterans with CKD Data represented as frequencies (n) and percentages (%)

Variable	Unweighted Frequency (n=3332)	Unweighted Percentage (%)	Weighted Frequency (n=26043538.5)	Weighted Percentage (%)
Sleep Duration				
1-5 hours	464	22.40%	22628	1.28%
6-10 hours	1746	71.83%	725675	4.10%
Above 10 hours	100	5.77%	58248	0.33%
Residence				
Rural	428	6.80%	92746	0.36%
Urban	2852	93.20%	1271693	4.93%
Alcohol Consumption				
No	1772	55.03%	689548	3.00%
Yes	1283	45.00%	563387	2.41%
Age Group				
18-24 years	12	1.14%	15672	0.06%
25-34 years	28	3.51%	48173	0.19%
35-44 years	64	3.92%	53822	0.20%
45-54 years	170	7.19%	98648	0.38%
55-64 years	393	13.30%	182549	0.70%
65 or older	2665	70.93%	973510	3.80%
Gender				
Female	304	11.09%	152238	0.59%
Male	3028	88.91%	1220135	4.70%
Race				
White	2725	70.67%	969869	3.74%
Black	252	14.61%	200524	0.77%
Hispanic	160	9.30%	128756	0.50%
American Indian	46	1.50%	20529	0.08%
Asian	41	1.18%	16143	0.06%
Other races	108	2.66%	36552	1.14%
Insurance				
Did not have insurance	27	1.69%	22228	0.09%
Had insurance	3192	98.31%	1296494	5.20%
Coronary Artery Disease				
Absent	2182	67.93%	913463	3.57%
Present	1060	32.07%	431256	1.68%
Diabetes Comorbidity				
Absent	1827	54.29%	742483	2.90%
Present	1497	45.71%	625174	2.42%
Stroke Comorbidity				
Absent	2799	80.13%	1094446	4.24%
present	508	19.87%	271461	1.05%
Depression Comorbidity				
Absent	2530	71.04%	962652	3.74%
Present	779	28.96%	392483	1.53%

Bivariate analysis

There were significant observations in the bivariate analysis. Veterans who slept for 6-10 hours had 10% lower unadjusted odds of developing CKD compared to those who slept for only 1-5 hours (OR=0.90, 95%CI= 0.904-0.913). Those who resided in the urban areas also had higher unadjusted odds of developing CKD compared to veterans living in rural areas. Veterans who were 65 years and older had 41% higher unadjusted odds of developing CKD (OR =5.923, 95%CI= 5.872-5.976). Veterans who were of Black race had higher unadjusted odds of developing CKD compared to veterans of the non-Hispanic race (OR= 1.032, 95%CI= 1.027-1.037, P <0001). The insurance variable was left out of the final model because it did not meet the 80% confidence benchmark after bivariate logistic regression.

Multivariate analysis

At the multivariate level, veterans with sleep duration of 6-10 hours had 17.5% lower odds of having CKD as compared to veterans who slept for 1-5 hours (adjusted OR (AOR)= 0.825, 95%CI= 0.821-0.830, P=<0.0001). Veterans who slept for more than 10 hours had 68.2% higher odds of having CKD as compared to veterans who slept for 1-5 hours (AOR=1.682, 95%CI= 1.662-1.702, P=<0.0001). Male veterans had 24.7% lower odds of having CKD as compared to female veterans. Veterans who consumed alcohol had 7.8% lower odds of having CKD as compared to individuals who did not consume alcohol (AOR= 0.922 CI =0.918,0.927 p=<0.0001). Veterans who are 65 years and older had higher odds of developing CKD compared to those aged 35-44 years (AOR= 5.743, 95%CI = 5.669-5.818, P<0.001). Furthermore, veterans diagnosed with diabetes, stroke, and CAD had higher odds of developing CKD (2.447, 2.103, and 2.838, respectively, 95%CI= 2.435-2.459, p=<0.0001). The combined bivariate and multivariate analysis breakdown is displayed in Table 2.

**Table 2:** Multivariate analysis of independent variable and all covariates The significant level set at p<0.05 Ref: reference variable; CI: confidence interval

Variables	Unadjusted			Adjusted		
	Odds Ratio	95% CI	P value	Odds Ratio	95% CI	P value
Sleep Duration						
1-5 hours	Ref (0.00)	Ref (0.00)	Ref (0.00)	Ref (0.00)	Ref (0.00)	Ref (0.00)
6-10 hours	0.909	(0.904, 0.913)	<0.0001	0.825	(0.821, 0.830)	<0.0001
Above 10 hours	3.464	(3.430, 3.499)		1.682	(1.662, 1.702)	
Residence						
Rural	ref	ref	ref	ref	ref	ref
Urban	1.013	(1.006, 1.020)	0.0003	1.271	(1.259, 1.282)	<0.0001
Gender						
Male	1.213	(1.206, 1.220)	<0.0001	0.753	(0.747, 0.758)	<0.0001
Female	ref	ref	ref	ref	ref	ref
Age Group						
18-24 years	0.622	(0.611, 0.634)	<0.0001	1.001	(0.979, 1.024)	0.9094
25-34 years	0.901	(0.889, 0.912)		1.16	(1.141, 1.179)	<0.1
35-44 years	ref	ref	ref	ref	Ref	ref
45-54 years	1.755	(1.737, 1.774)	<0.001	2.019	(1.990, 2.049)	<0.1
55-64 years	2.737	(2.710, 2.764)	<0.001	2.797	(2.759, 2.835)	
65 or older	5.923	(5.872, 5.976)	<0.03	5.743	(5.669, 5.818)	<0.001
Race						
White	ref	ref	ref	ref	ref	ref
Black	1.032	(1.027, 1.037)	<0.0001	1.397	(1.388, 1.405)	<0.0001
Hispanic	0.842	(0.837, 0.847)	<0.0003	1.318	(1.307, 1.329)	<0.001
Asian	0.465	(0.457, 0.472)	<0.001	0.87	(0.853, 0.888)	<0.01
Other races	0.591	(0.585, 0.597)	<0.001	0.609	(0.600, 0.618)	<0.001
American Indian	0.925	(0.912, 0.938)	<0.001	1.299	(1.279, 1.319)	
Alcohol Consumption						
No	ref	ref	ref	ref	ref	ref
Yes	0.617	(0.614, 0.619)	<0.0001	0.922	(0.918, 0.927)	<0.0001
CAD Comorbidity						
Absent	ref	ref	ref	ref	ref	ref
Present	5.506	(5.485, 5.528)	<0.001	2.834	(2.819, 2.849)	<0.0001
Diabetes Comorbidity						
Absent	ref	ref	ref	ref	ref	Ref
Present	4.539	(4.523, 4.555)	<0.001	2.447	(2.435, 2.459)	<0.0001
Stroke Comorbidity						
Absent	ref	ref	ref	ref	ref	ref
Present	4.278	(4.259, 4.298)	<0.0001	2.103	(2.089, 2.116)	<0.0001
Depression Comorbidity						
Absent	ref	ref	ref	ref	Ref	Ref
Present	1.724	(1.718, 1.731)	<0.0001	1.571	(1.563, 1.580)	<0.0001

## Discussion

Based on data from the BRFSS, this study considered CKD based on self-reported prior diagnoses of kidney disease. The demographic statistics of this study of veterans with CKD align with the 2022 US VA enrollment data, indicating a higher enrollment percentage among individuals aged 65 years and above (47.8%) and an approximate male-to-female ratio of 15:2 among enrolled veterans [44]. The findings of this study indicated the prevalence of CKD among veterans to be 6.29%, which aligns with findings from a 2018 study reporting a period prevalence of CKD among veterans at 6.1% [45]. However, there is an uptick from a 2015 survey of the Military Health System database, which indicated a CKD prevalence of 5.4% among non-active duty individuals [7]. 

The prevalence of CKD after controlling for other variables is elevated among veterans with both short sleep duration (1-5 hours of sleep in 24 hours) and prolonged sleep duration (>10 hours of sleep in 24 hours). This finding is consistent with a cross-sectional analysis conducted by Jiang and colleagues [29], as well as an observational cohort study conducted by Park et al. [30]. Contrary to findings from two previous nonveteran studies on demographic CKD prevalence data [46,47], the current study observed that veterans who were urban dwellers had a higher incidence of CKD compared to rural dwellers. The fast-paced lifestyle and noise associated with city living can indeed disrupt sleep patterns and, consequently, have a negative impact on overall health. When stratifying for age, the prevalence of CKD was observed to increase as the age of veterans increased. This trend is consistent with a previous study that identified age as a significant risk factor for both the prevalence and progression of CKD [48]. More so, CKD can arise as a complication of poorly controlled diabetes and hypertension comorbidities, both of which also tend to increase in prevalence with age.

Examining gender association in this study, there is an increased prevalence of CKD among female veterans compared to males, aligning partially with findings from Oliver et al.'s retrospective cohort study, which reported an overall higher prevalence of CKD specifically among females in active duty compared to males. However, Haroun et al.'s prospective analysis highlighted the association of CKD occurrence in women with both early and late stages of hypertensive comorbidity, compared to CKD prevalence in men, which was associated only with later stages of hypertensive comorbidity [49]. Furthermore, Veterans who identify as Black, Hispanic, and American Indian races demonstrated a higher prevalence of CKD compared to those who identify as non-Hispanic White and Asian races. This observation aligns with the findings of Sheehan et al., who emphasized the exacerbating disparities in sleep duration and its correlation with deteriorating overall health outcomes among minority populations [18]. Another interesting finding from our analysis is the negative association between alcohol consumption and the prevalence of CKD. This result is in tandem with findings from Koning et al. [50]; however, it is important for future experimental studies to further elucidate the relationship between different measures of alcohol consumption and their effects on kidney function.

Strengths and limitations

This study has several strengths worth noting. Firstly, it benefits from a large sample size, which enhances the reliability and generalizability of the findings. Additionally, the demographic data obtained from the BRFSS serves as a microcosm of VA enrollment, providing valuable insights into the veteran population. Furthermore, the study meticulously accounted for various confounding variables, ensuring a robust investigation into the association between sleep duration and CKD prevalence among US veterans.

This study had the following limitations. First, demographic, behavioral, and health status data were all self-reported, which gives rise to recall and social desirability biases [12,18]. For example, the BRFSS question on sleep “On average, how many hours of sleep do you get in 24 hours?” is unspecific, and responses were restricted to whole number values giving rise to a possible misclassification from recent sleep pattern changes and problems with recall. Utilizing a sleep diary [35] or actigraphy and polysomnography for future studies would give a more reliable assessment of the length of sleep [18,20,51]. Second, veteran status recorded in the BRFSS does not provide detailed information on past military experiences, such as length of service, disability status, time elapsed between deployment and survey participation, severity of injuries, and prior combat locations [20,23]. These factors could potentially create additional categories that would further explain the strength of the association between sleep duration and CKD among veterans. Therefore, future studies investigating the relationship between sleep and CKD among veterans should seek out databases that include these specific veteran variables. Finally, this study utilized snapshot data from the 2022 BRFSS survey to explore the relationship between sleep duration and the prevalence of CKD. However, this limits our ability to establish causality between sleep duration and CKD. Nevertheless, short and long sleep duration has been shown to correlate well with increased prevalence of CKD.

## Conclusions

Although comorbidities such as heart disease and diabetes mellitus are well-established risk factors for CKD, sleep duration has not received similar recognition. This study sheds light on the significance of sleep duration as another important factor in addressing the CKD epidemic among veterans. Public health initiatives aimed at promoting optimal sleep should be prioritized early in military recruitment processes to mitigate the risk of CKD development.
